# Community-acquired *Acinetobacter baumannii* pneumonia in Southern Vietnam: A retrospective cohort study

**DOI:** 10.1097/MD.0000000000050995

**Published:** 2026-10-09

**Authors:** Thu-Huong Le-Thi, Quoc-Khanh Tran-Le, Lam Nguyen-Ho, Bao Tran-Gia, Hang Nguyen-Thi-Thuy, Phuong Huynh-Thi-Thanh, Nguyen Tien Huy, Tuong Tran-Thi-Thuy

**Affiliations:** a Respiratory Department, Nhan Dan Gia Dinh Hospital, Ho Chi Minh City, Vietnam; b Internal Medicine Department, School of Medicine, University of Medicine and Pharmacy at Ho Chi Minh City, Ho Chi Minh City, Vietnam; c Respiratory Functional Exploration Department, University Medical Center HCMC, University of Medicine and Pharmacy at Ho Chi Minh City, Ho Chi Minh City, Vietnam; d Infection Control Department, Nhan Dan Gia Dinh Hospital, Ho Chi Minh City, Vietnam; e Institute of Research and Development, Duy Tan University, Da Nang, Vietnam; f School of Medicine and Pharmacy, Duy Tan University, Da Nang, Vietnam; g School of Tropical Medicine and Global Health (TMGH), Nagasaki University, Nagasaki, Japan.

**Keywords:** *Acinetobacter baumannii*, antimicrobial resistance, community-acquired pneumonia, outcomes, Vietnam

## Abstract

*Acinetobacter baumannii* has traditionally been considered a nosocomial pathogen, but cases of community-acquired *A. baumannii* pneumonia have increasingly been reported in tropical Asia, including Vietnam. Data on its clinical characteristics, antimicrobial resistance, and patient outcomes remain limited. This study aimed to describe the clinical profile, resistance patterns, and outcomes of community-acquired A. baumannii pneumonia in southern Vietnam. We performed a single-center retrospective cohort study at Nhan Dan Gia Dinh Hospital, a 1500-bed tertiary center in Ho Chi Minh City (January 2021-December 2023). Adults (≥18 years) with community-acquired pneumonia and a positive sputum, endotracheal aspirate, or blood culture for *A. baumannii* obtained within 48 hours of admission were included. Patients with cultures >48 hours after admission, hospitalization in the prior 90 days, residence in/transfer from another facility ≥48 hours preadmission, polymicrobial cultures, or incomplete records were excluded. Thirty-one patients were identified (median age 69.0 years (interquartile range 64.0−84.0); 83.9% male; 83.9% current/former smokers). No clear seasonal predominance was observed within the study window after month-level normalization. On admission, 87.1% had hypoxemia (peripheral oxygen saturation<90%) and nearly half (45.2%) were severe community-acquired pneumonia. Antibiotic escalation was required in 61.3% after a median of 4 days (interquartile range 3−5). Resistance was approximately 69%–83% across the routinely tested β-lactams and fluoroquinolones, whereas aminoglycosides showed comparatively higher in vitro activity than the other tested antibiotic classes but susceptibility remained suboptimal (amikacin 50%, tobramycin 46%, gentamicin 36%). Initial empiric therapy was active against the index isolate in 4/25 patients (16.0%) with available susceptibility results. In-hospital mortality was 13/31 (41.9%). Community-*acquired* A. baumannii pneumonia is associated with severe disease, extensive antimicrobial resistance, frequent empiric treatment mismatch, and high mortality. In resource-constrained settings, limited therapeutic options highlight the importance of early microbiological diagnosis, comprehensive susceptibility testing, and locally informed treatment strategies.

## 1. Introduction

*Acinetobacter baumannii* is a Gram-negative coccobacillus that has long been recognized as a common cause of hospital-acquired infections, especially ventilator-associated pneumonia. However, over the past 2 decades, a growing body of evidence has identified *A. baumannii* as an etiologic agent in community-acquired pneumonia (CAP), particularly in tropical and subtropical regions.^[1–4]^

Community-acquired A. baumannii (CA-AB) pneumonia typically affects older adults and individuals with chronic comorbidities (e.g., chronic lung disease, diabetes), often in association with tobacco or hazardous alcohol use.^[1,5,6]^ Published cohorts of CA-AB pneumonia, predominantly from tropical/subtropical regions, consistently describe a fulminant phenotype with frequent shock, low rates of initially active empiric therapy, and substantial mortality.^[1–3]^ Reported seasonality varies by setting, with some series showing wet-season predominance^[1,7,8]^ and others reporting cases year-round.^[3]^ Importantly, guideline-concordant empirical regimens for CAP^[9]^ frequently lack activity against *A. baumannii*, leading to delays in appropriate therapy.^[2]^ Despite growing recognition of CA-AB pneumonia in Asia, contemporary data from Vietnam remain limited. We therefore conducted a single-center retrospective cohort study at Nhan Dan Gia Dinh Hospital (Ho Chi Minh City) to describe the clinical presentation, seasonality, antimicrobial susceptibility patterns, adequacy of initial empirical therapy, and outcomes among adults with CA-AB pneumonia. Defining local patterns can inform empiric antibiotic guidance and may improve outcomes for this pathogen.

## 2. Materials and methods

### 2.1. Study design and setting

We conducted a single-center, retrospective cohort study of adults hospitalized with CA-AB pneumonia at Nhan Dan Gia Dinh Hospital, a 1500-bed tertiary-care center in Ho Chi Minh City, which serves as a referral center for southern Vietnam. All eligible admissions between 1 January, 2021 and 31 December, 2023 were included.

This manuscript was prepared in accordance with the Strengthening the Reporting of Observational Studies in Epidemiology guidelines for cohort studies.^[10]^ Given the retrospective identification of eligible patients and the comparison of clinical characteristics and outcomes between survivors and non-survivors, the study was considered a retrospective cohort study. This study was conducted in accordance with the Declaration of Helsinki and was approved by the Institutional Ethics Committee of Nhan Dan Gia Dinh Hospital (approval no. 91/NDGĐ-HĐĐĐ). The requirement for informed consent was waived because of the retrospective nature of the study. All data were anonymized prior to analysis.

A subset of patients with severe CAP from this cohort was previously reported by Tran TTT et al^[11]^; the present study expands upon that work by analyzing the full cohort of CA-AB pneumonia cases.

### 2.2. Patient selection

We conducted a retrospective review of the microbiology database at Nhan Dan Gia Dinh Hospital from January 1, 2021, to December 30, 2023, to identify all patients with *Acinetobacter baumannii* isolated from respiratory (sputum or endotracheal aspirate) or blood cultures. From this cohort, we selected patients diagnosed with CAP. Pneumonia was diagnosed according to the Vietnamese Ministry of Health guidelines, requiring the presence of compatible clinical symptoms (e.g., fever, cough, sputum production, dyspnea) combined with a new pulmonary infiltrate on chest radiograph or computed tomography at presentation.^[12]^ We defined CAP as pneumonia with symptom onset in the community or within the first 48 hours after hospital admission; the microbiological culture (blood and/or respiratory specimen) was obtained within 48 hours of admission. Hospital-acquired pneumonia was defined as pneumonia with onset ≥48 hours after admission and was excluded. To ensure the community-acquired nature of the infections, we excluded patients with documented healthcare-associated exposures. Specifically, we excluded individuals who had a hospitalization within the preceding 90 days, those residing in or transferred from another healthcare facility for ≥48 hours immediately prior to admission, and patients receiving hemodialysis, chemotherapy, or specialized wound care. Cases were defined as monomicrobial *A. baumannii* pneumonia; polymicrobial cultures in which *A. baumannii* co-occurred with another significant bacterial pathogen were excluded to ensure clinical features were attributable to *A. baumannii*. We also excluded patients with incomplete records or missing key clinical outcome data. For patients with multiple eligible encounters, only the first admission and the earliest qualifying specimen were included in the analysis.

### 2.3. Specimen collection and A. baumannii detection methods

Sputum was collected within 48 hours of admission, prior to antibiotics when feasible, into sterile containers. Samples were then transported promptly and processed within 2 hours, or stored at 2° to 8°C and processed within 24 hours. The adequacy of sputum specimens was assessed microscopically using Bartlett’s count/grading criteria. Briefly, an optimum sputum sample was defined as one with minimal oropharyngeal contamination and evidence of lower-respiratory inflammation, corresponding to <10 squamous epithelial cells and ≥25 polymorphonuclear leukocytes per low-power field, equivalent to an acceptable Bartlett score.^[13]^ Specimens not meeting these criteria were considered poor-quality samples and were not used as qualifying respiratory specimens for case inclusion. Endotracheal aspirates obtained within 48 hours of admission were handled identically.

Presumptive Acinetobacter isolates were screened by Gram staining, which showed Gram-negative, non-motile coccobacilli, typically isolated singly or in pairs.^[4]^ After 18 to 24 hours of incubation at 35°C, colonies appeared as smooth, non-lactose-fermenting growth on MacConkey agar and were catalase-positive, oxidase-negative, features consistent with the *Acinetobacter* genus.^[14]^ Species identification was performed using the VITEK® 2 system (bioMérieux, Marcy-l’Étoile, France) and confirmed by matrix-assisted laser desorption/ionization time-of-flight mass spectrometry (Smart MS 5020, Zhuhai DL Biotech Co., Ltd., Zhuhai, China). Antimicrobial susceptibility testing was carried out on the VITEK® 2 platform using the GN ID card and relevant antimicrobial susceptibility testing-GN susceptibility cards, and interpretive criteria were based on the Clinical and Laboratory Standards Institute document M100.^[15]^

Blood cultures collected within 48 hours of admission were inoculated into paired aerobic and anaerobic bottles and incubated in an automated continuous-monitoring blood culture system (BACTEC™ or BacT/ALERT®) for up to 5 days, depending on instrument availability in our hospital microbiology laboratory during the study period.

Evaluated antimicrobial agents included third- and fourth-generation cephalosporins, *β*-lactam/*β*-lactamase inhibitor combinations, carbapenems, fluoroquinolones, aminoglycosides, and trimethoprim/sulfamethoxazole. Polymyxins and newer agents (tigecycline/minocycline, cefiderocol, sulbactam-durlobactam) were not recorded, as they were not routinely included in the susceptibility testing panel for community isolates at our institution during the study period.

### 2.4. Data collection

Data were abstracted from the electronic medical record system using a standardized collection form. Variables collected included demographics (age, sex), smoking status, comorbidities (hypertension; chronic lung disease, including chronic obstructive pulmonary disease, asthma, and bronchiectasis; and diabetes), presenting vital signs (peripheral oxygen saturation, respiratory rate, and blood pressure), laboratory values (white blood cell count and C-reactive protein [CRP]), radiologic findings, and clinical outcomes (development of shock, need for invasive mechanical ventilation, and in-hospital mortality). Antimicrobial therapy, including initial regimen, dose, and modification during treatment, was also recorded.

### 2.5. Statistical analysis

Statistical analyses were conducted with SPSS version 25 (IBM Corp., Armonk). Continuous variables were tested for normality with the Shapiro–Wilk test and presented as mean ± standard deviation or median with interquartile range, as appropriate. Categorical variables were expressed as number and percentage. The Student *t* test or Mann-Whitney *U* test compared continuous variables, whereas the *χ*^*2*^ or Fisher’s exact test compared categorical variables. A 2-tailed *P* < .05 was considered statistically significant. Given the small sample size and the number of comparisons, comparisons between survivors and non-survivors were considered exploratory and hypothesis-generating; no adjustment for multiple testing was performed. Variables reflecting events during hospitalization (e.g., mechanical ventilation and septic shock) were summarized descriptively and were not interpreted as baseline predictors of mortality. Seasonal case rates were compared using a two-sample Poisson rate score test, with the number of months in each season treated as the exposure.

## 3. Results

### 3.1. Patient characteristics

Thirty-one patients were included in the final analysis (Fig. 1). The cohort comprised predominantly older adults (median age 69.0 years, interquartile range [IQR] 64.0–84.0); 23/31 (74.2%) were aged ≥65 years. Twenty-six patients (83.9%) were male, and 26/31 (83.9%) were current or former smokers. Comorbidities were common: hypertension (26/31, 83.9%), chronic lung disease (18/31, 58.1%), and diabetes mellitus (12/31, 38.7%) (Table 1). Patients with CA-AB pneumonia presented with severe manifestations. On admission, 27/31 (87.1%) were hypoxemic (peripheral oxygen saturation <90%), 2/31 (6.5%) required vasopressor support, and 8/31 (25.8%) were admitted directly to the respiratory intensive care unit (RICU).

**Table 1:** Clinical characteristics of patients with community-accquired pneumonia due to *A. baumannii* (n = 31).

	Overall (n = 31)	Survivors (n = 18)	Non-survivors (n = 13)	*P*-value
Demographics				
Age, yr, median (IQR)	69.0 (64.0–84.0)	71.5 (64.0–84.0)	69.0 (66.0–75.0)	.929
Male, n (%)	26 (83.9%)	14 (77.8%)	12 (92.3%)	.278
Comorbidities, n (%)				
Diabetes mellitus	12 (38.7%)	7 (38.9%)	5 (38.5%)	.981
Heart failure	5 (16.1%)	2 (11.1%)	3 (23.1%)	.625
Chronic lung disease	18 (58.1%)	12 (66.7%)	6 (46.2%)	.253
Chronic kidney disease	0 (0.0%)	0 (0.0%)	0 (0.0%)	
Hypertension	26 (83.9%)	17 (94.4%)	9 (69.2%)	.134
History of cerebrovascular accident	7 (22.6%)	3 (16.7%)	4 (30.8%)	.618
Current or former smoker, n (%)	26 (83.9%)	13(72.2%)	13(100.0%)	.058
Clinical presentation, n (%)				
Hypoxemia (SpO_2_ < 90%)	27 (87.1%)	15 (83.3%)	12(92.3%)	.621
Vasopressor use on admission	2 (6.5%)	0 (0.0%)	2 (15.4%)	.168
RICU admission on first day	8 (25.8%)	2 (11.1%)	6 (46.2%)	**.043**
Acute kidney injury on admission	3 (9.7%)	1 (5.6%)	2 (15.4%)	.558
Laboratory findings, median (IQR)				
WBC (×10^9^/L)	13.6 (9.6–18.5)	14.1 (9.1–17.6)	12.0 (10.3 –19.9)	.567
Neutrophils (×10^9^/L)	11.5 (6.2–16.5)	12.9 (6.1–15.0)	10.2 (8.1–17.2)	.594
Hemoglobin (g/L)	122.0 (108.0–138.0)	130.5 (115.0–139.0)	110.0 (107.0–135.0)	.143
Platelets (×10^9^/L)	261.0 (170.0–344.0)	254.5 (207.0–306.0)	263.0 (168.0–445.0)	.828
AST (U/L)	41 (20.8–54.7)	28.5 (20.4–45.3)	45.0 (25.0–54.7)	.435
ALT (U/L)	22.4 (14.5–46.0)	27.8 (16.6–47.0)	20.4 (15.4–41.1)	.458
Creatinine (µmol/L)	94.4 (68.9–113.0)	80.1 (70.5–104.9)	102.0 (68.9–119.2)	.327
CRP (mg/L)	90 (51.8–141.0)	64.6 (30.0–93.5)	129.5 (99.1–155.0)	**.010**
Radiologic findings, n (%)				
Localized infiltration	19 (61.3%)	13 (72.2%)	6 (46.2%)	.141
Diffuse infiltration	12 (38.7%)	5 (27.8%)	7 (53.9%)	
Microbiology, n (%)				
Positive sputum culture only	26 (83.9%)	17 (94.4%)	9 (69.2%)	.064
Positive blood culture only	2 (6.5%)	1 (5.6%)	1 (7.7%)	
Both sputum & blood cultures positive	3 (9.7%)	0 (0.0%)	3 (23.1%)	
Severe pneumonia on admission, n (%) (95% CI)	14 (45.2%, 27.3–64.0)	2 (11.1%, 1.4–34.7)	12 (92.3%, 64.0–99.8)	**<.001**
Outcomes, n (%) (95% CI)				
Antibiotic escalation	19 (61.3%, 42.2–78.2)	9 (50.0%, 26.1–74.0)	10 (76.9%, 46.2–95.0)	.126
Mechanical ventilation	13 (41.9%, 24.5–60.9)	2 (11.1%, 1.4–34.7)	11 (84.6%, 54.6–98.1)	**<.001**
Septic shock	12 (38.7%, 21.8–57.8)	1 (5.6%, 0.1–27.3)	11 (84.6%, 54.6–98.1)	**<.001**

Proportion 95% CIs are Clopper–Pearson exact.

Mann–Whitney *U* test for continuous variables; *χ*^2^ or Fisher’s exact test for categorical variables, as appropriate.

ALT = alanine aminotransferase, AST = aspartate aminotransferase, CI = confidence interval, CRP = C-reactive protein, IQR = interquartile range, RICU = respiratory intensive-care unit, SpO_2_ = peripheral oxygen saturation, WBC =  white-blood-cell count.

**Figure 1.**
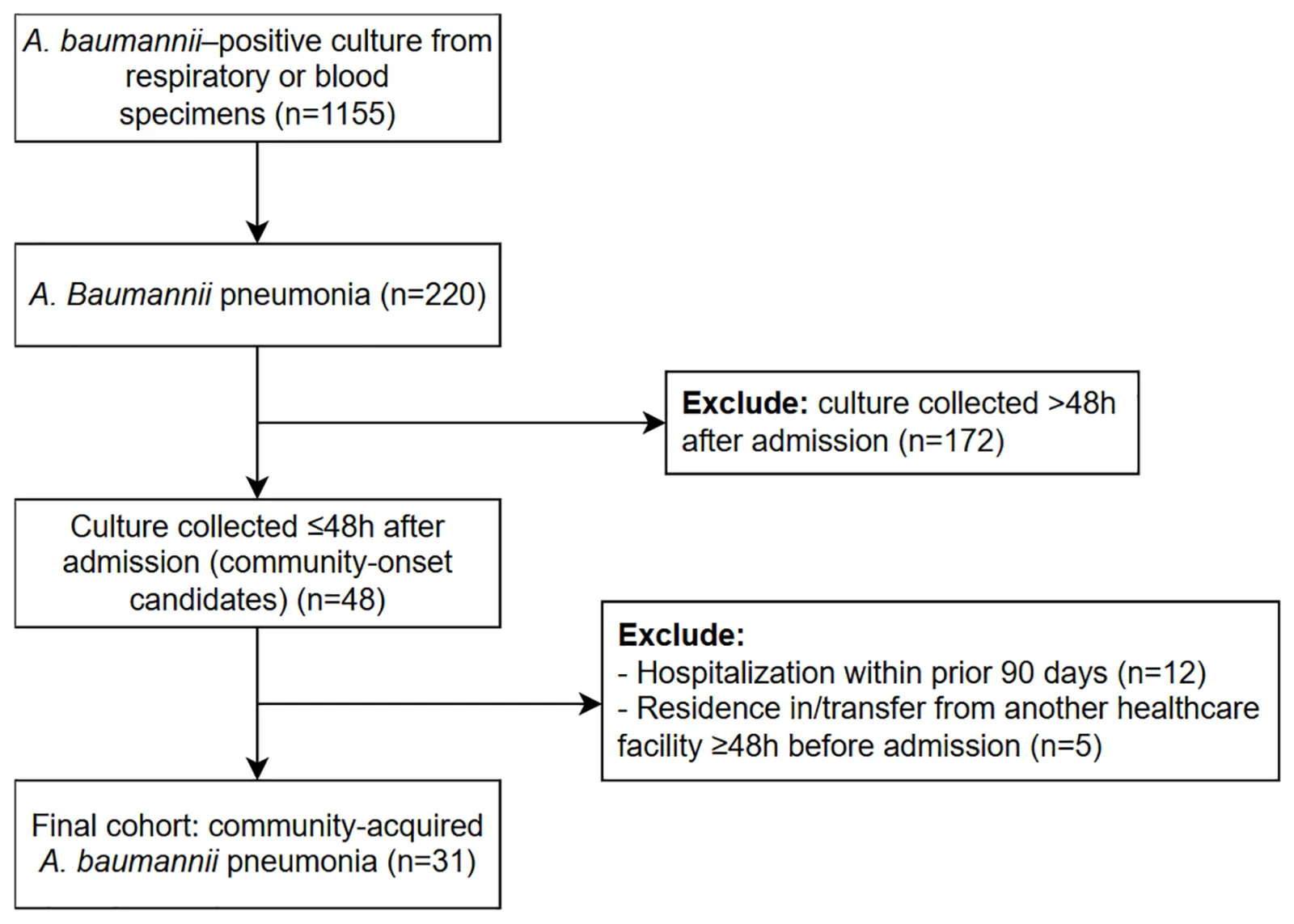
Flowchart of patient selection.

Systemic inflammation was evident (Table 1): median white blood cell count 13.6 × 10^9^/L (IQR 9.6−18.5) with increased neutrophils (11.5 × 10^9^/L [6.2−16.5]). Median CRP was 90 mg/L (IQR 51.8−141). Chest radiographs showed lobar consolidation in 19/31 patients (61.3%) and bilateral or multilobar infiltrates in 12/31 (38.7%).

*A. baumannii* was most frequently isolated from sputum samples (29/31, 93.5%). Bloodstream infection was identified in 5 patients (5/31, 16.1%), of whom 3 had concordant positive cultures from both sputum and blood. During the study period (January 2021–December 2023), we did not observe a clear seasonal pattern in CA-AB pneumonia. A total of 16 cases (51.6%) were recorded during the dry season (December-April) compared to 15 cases (48.4%) during the rainy season (May-November).

After accounting for the unequal number of dry- and rainy-season months within the study period (15 vs 21 months), the monthly case rates were 1.07 and 0.71 cases/month, respectively (*P* = .261).

### 3.2. Clinical outcomes

Fourteen patients (14/31, 45.2%; 95% confidence interval (CI) 27.3−64.0) met the 2007 American Thoracic Society (ATS)/Infectious Diseases Society of America (IDSA) criteria for severe CAP at presentation (Table 1). Thirteen patients (13/31, 41.9%; 95% CI 24.5−60.9) required invasive mechanical ventilation; 12 (12/31, 38.7%; 95% CI 21.8−57.8) developed septic shock; and 19 (19/31, 61.3%; 95% CI 42.2−78.2) underwent antibiotic escalation. Overall, in-hospital mortality was 13/31 (41.9%; 95% CI 24.5−60.9).

Patients were classified as survivors (*n *= 18) or non-survivors (*n* = 13). Baseline age, sex, and comorbidities did not statistically differ. On admission, non-survivors were more often admitted directly to the RICU (6/13, 46.2% vs 2/18, 11.1%; *P* = .043). They also showed a stronger systemic inflammatory response, with CRP nearly double that of survivors (129.5 (99.1−155) mg/L vs 64.6 (30−93.5) mg/L; *P* = .010); other laboratory indices were similar. Median hospital stay was 9 days (IQR, 7−17) and did not differ between groups (*P* = .355).

### 3.3. Antibiotic use and resistance

Empiric therapy consisted of 2-drug regimens in 24/31 (77.4%), 3-drug regimens in 5/31 (16.1%), and monotherapy in 2/31 (6.5%). Most regimens paired a β-lactam with either a macrolide or a fluoroquinolone. Third-generation cephalosporins (predominantly ceftriaxone) were used in 8/31 (25.8%); *β*-lactam/*β*-lactamase inhibitor combinations (piperacillin-tazobactam or cefoperazone-sulbactam) in 12/31 (38.7%); and carbapenems (imipenem-cilastatin or meropenem) in 10/31 (32.3%). The companion agent was most often a fluoroquinolone in 23/31 (74.2%), usually levofloxacin, or a macrolide (clarithromycin) in 5/31 (16.1%). Initial antimicrobial regimens are detailed in Table 2.

**Table 2:** Initial antimicrobial regimens prescribed for patients with community-acquired *Acinetobacter baumannii* pneumonia (n = 31).

First-line antimicrobial therapy administered	n (%)
Piperacillin/Tazobactam + Levofloxacin	6 (19.4)
Piperacillin/Tazobactam + Levofloxacin + Vancomycin	1 (3.2)
Piperacillin/Tazobactam + Clarithromycin	1 (3.2)
Piperacillin/Tazobactam + Ciprofloxacin	1 (3.2)
Piperacillin/Tazobactam + Ciprofloxacin + Vancomycin	1 (3.2)
Levofloxacin	1 (3.2)
Meropenem + Vancomycin	1 (3.2)
Imipenem/Cilastatin + Vancomycin	1 (3.2)
Imipenem/Cilastatin + Levofloxacin + Amikacin	1 (3.2)
Imipenem/Cilastatin + Levofloxacin	3 (9.7)
Imipenem/Cilastatin + Levofloxacin + Vancomycin	2 (6.5)
Imipenem/Cilastatin + Ciprofloxacin	2 (6.5)
Ceftriaxone + Levofloxacin	4 (12.9)
Ceftriaxone + Clarithromycin	2 (6.5)
Ceftriaxone	1 (3.2)
Ceftazidime + Clarithromycin	1 (3.2)
Cefoperazone/sulbactam + Levofloxacin	1 (3.2)
Cefoperazone/sulbactam + Clarithromycin	1 (3.2)

Among cases with available antibiograms, empiric therapy was active against the *A. baumannii* index isolate in only 4/25 (16.0%), indicating low adequacy of initial empiric therapy. Overall, 19/31 (61.3%) required escalation to broader or additional agents after a median of 4 days (IQR 3-5) due to inadequate clinical response.

Antimicrobial susceptibility results for at least one evaluated agent were available for 25/31 isolates (80.6%). Resistance was high among the routinely tested β-lactams and fluoroquinolones. Resistance to third- and fourth-generation cephalosporins was observed in 16/20 (80.0%) isolates tested for ceftriaxone and 19/24 (79.2%) each for ceftazidime and cefepime. Among *β*-lactam/*β*-lactamase inhibitor combinations, 14/20 (70.0%) isolates were resistant to ampicillin–sulbactam and 19/23 (82.6%) to piperacillin–tazobactam. Carbapenem resistance was observed in 17/24 (70.8%) isolates tested for imipenem–cilastatin and 9/13 (69.2%) for meropenem. Resistance to fluoroquinolones was also high, occurring in 19/25 (76.0%) isolates tested for ciprofloxacin and 19/24 (79.2%) for levofloxacin. Aminoglycosides demonstrated comparatively greater in vitro activity, although susceptibility remained suboptimal: amikacin 8/16 (50.0%), tobramycin 11/24 (45.8%), and gentamicin 9/25 (36.0%). These susceptibility findings are summarized in Figure 2.

**Figure 2.**
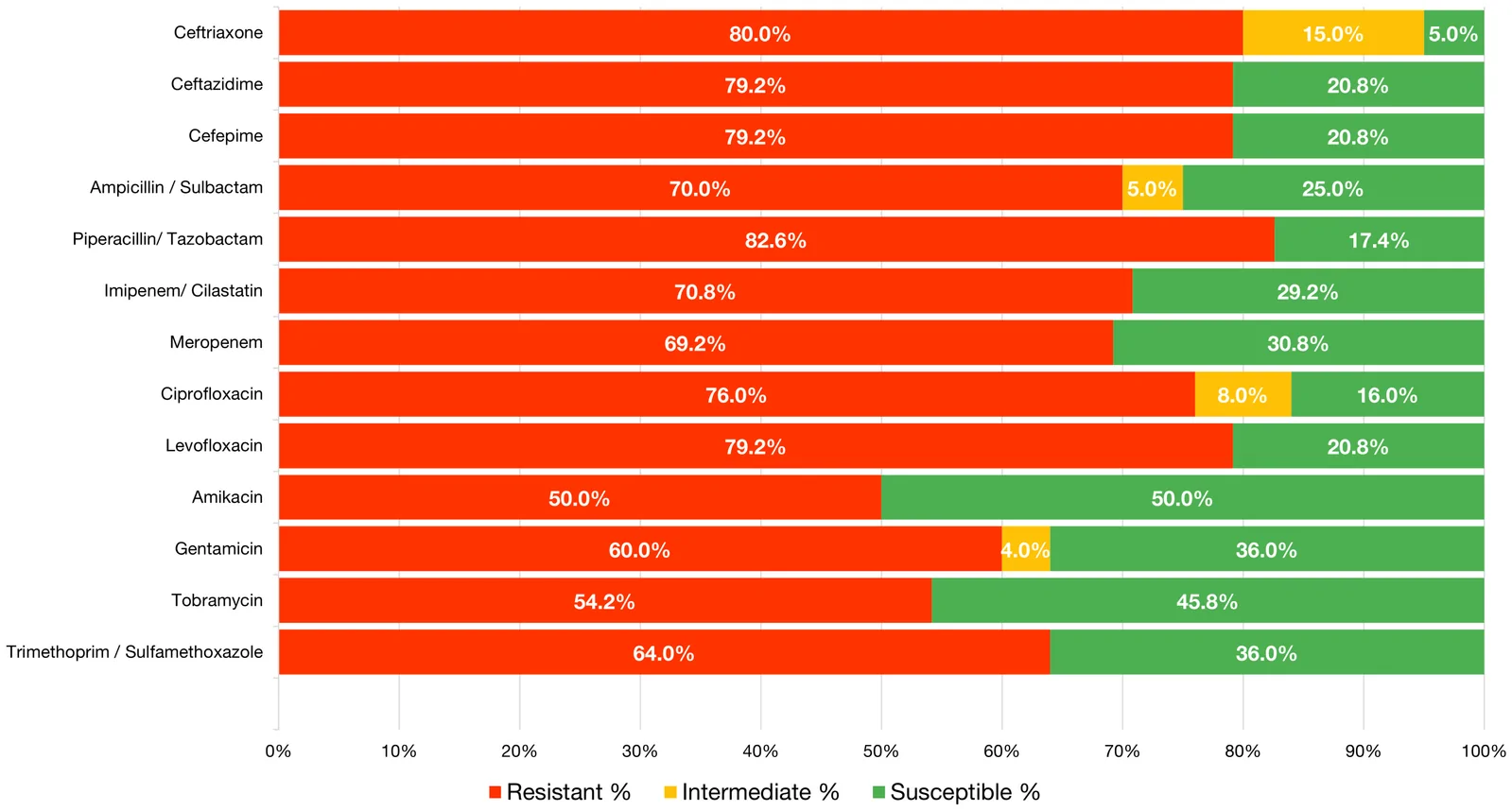
In vitro antimicrobial susceptibility profile of community-acquired Acinetobacter baumannii isolates. Percentages were calculated using the number of isolates with an available susceptibility result for each antimicrobial agent as the denominator; untested or missing results were excluded. Numbers tested were: ceftriaxone (n = 20), ceftazidime (n = 24), cefepime (n = 24), ampicillin/sulbactam (n = 20), piperacillin/tazobactam (n = 23), imipenem/cilastatin (n = 24), meropenem (n = 13), ciprofloxacin (n = 25), levofloxacin (n = 24), amikacin (n = 16), gentamicin (n = 25), tobramycin (n = 24), and trimethoprim/sulfamethoxazole (n = 25).

## 4. Discussion

CA-AB pneumonia remains rare globally. Large surveillance studies report a prevalence of <1% among culture-positive CAP cases,^[16]^ and endemic tropical settings have estimated incidences of approximately 0.6 to 1.8 cases per 100,000 person-years.^[7]^ Consequently, most published cohorts remain small even in endemic regions, typically accruing only 8 to 36 cases over prolonged observation^[1,3,6–8,17,18]^ (e.g., 36 cases over 16 years^[7]^; 28 cases over 20 years^[18]^). Against this background, our single-center series of 31 adults admitted in 2021-2023 provides contemporary, region-specific evidence with clear management implications. Our study adds to the evidence that *A. baumannii* should no longer be viewed only as a hospital-acquired pathogen in tropical settings. These findings therefore provide practical evidence for strengthening local surveillance, refining empirical treatment pathways, and supporting antimicrobial stewardship decisions. We observed a fulminant phenotype, older patients with multiple comorbidities, frequent severe CAP, and high in-hospital mortality (41.9%), together with a pronounced early “therapeutic gap.” Specifically, isolates showed extensive resistance with limited *β*-lactam and fluoroquinolone activity and comparatively higher but still suboptimal aminoglycoside susceptibility; initial empiric therapy was active in only 4/25 (16.0%) cases with available testing, prompting frequent escalation. Notably, cases occurred year-round without seasonal predominance, indicating that wet-season cues may not reliably identify risk in urban southern Vietnam. Collectively, these findings support earlier year-round recognition and locally informed empiric regimens, with rapid reassessment and de-escalation once data are available, to reduce delays to active therapy in high-risk presentations.

*A. baumannii* is a pleomorphic, non-motile, encapsulated, aerobic Gram-negative coccobacillus. Although carriage in healthy individuals is uncommon, asymptomatic oropharyngeal colonization has been documented in up to 7% of the general population,^[19]^ and around 10% throat carriage among individuals with hazardous alcohol use during the wet season,^[20]^ lending biological plausibility to a colonization-then-aspiration pathogenesis in susceptible hosts. Beyond humans, environmental recovery of *A. baumannii* outside hospitals has been inconsistent, detected sporadically on soil, vegetables, and public surfaces in some settings but absent in others, so the true community reservoir remains undefined.^[5,21]^ Even so, community-acquired infections, particularly pneumonia, continue to carry substantial mortality.^[18]^

Although *A. baumannii* is classically described as a nosocomial pathogen, multiple Asia-Pacific reports have established CA-AB pneumonia as a distinct, often fulminant syndrome with rapid progression, frequent bacteremia, and high mortality. Early cohorts from subtropical settings, including Taiwan,^[1]^ Hong Kong^[6]^ and Australia,^[17]^ highlighted severe disease and early deterioration (e.g., septic shock/ARDS). Subsequent case series from other tropical or semi-tropical regions, including Singapore^[2]^ and Thailand,^[3]^ reinforced the high lethality and the practical challenge of initially inactive CAP regimens. Our study from Vietnam adds contemporary, Vietnam-specific data and supports the view that CA-AB pneumonia occurs across multiple Southeast Asian settings rather than being restricted to a small number of locales.

To contextualize our findings, we conducted a focused literature review (Table 3) of PubMed, Embase, Web of Science, and Scopus using terms combining “*Acinetobacter baumannii*,” “pneumonia,” and ‘community-acquired/onset’; eligible studies reported community-acquired (or adjudicated community-onset) *A. baumannii* pneumonia with extractable cohort data, while case reports and purely hospital-acquired/ventilator-associated series were excluded. We identified 9 studies spanning Australia,^[7,17,18]^ Taiwan,^[1]^ Hong Kong,^[6]^ Singapore,^[2]^ Thailand,^[3]^ Réunion Island/France,^[8]^ and the United States.^[22]^

**Table 3:** Community-acquired *A. baumannii* pneumonia: comparative study characteristics, outcomes, and seasonality.

	Anstey NM (1981–1991)	Chen MZ (1993–1999)	Leung WS (2000–2003)	Ong CWM (2009)	Davis JS (1997–2012)	Patamatamkul S (2017)	Serota DP (2018)	Riddles (2000–2019)	Rotini G (2014–2022)	Our study (2021–2023)
Country	Australia	Taiwan	Hong Kong	Singapore	Australia	Thailand	USA	Australia	Réunion Island, France	Vietnam
Design	Retrospective case series	Retrospective case series (severe CAP)	Retrospective case-control (CA-AB vs HA-AB pneumonia)	Retrospective case series	Prospective observational cohort (community bacteremic AB pneumonia)	Case-control (CA-AB pneumonia vs melioidosis CAP)	Case series + literature review (North American CA-AB pneumonia)	Retrospective cohort (community-acquired, bacteremic Ab pneumonia)	Retrospective ICU case series (severe CA-AB pneumonia)	Retrospective cohort study
N	11	13	19	8	36	18	19	28	8	31
Age	56.0*	66.0†	72.6*	58.5†	44.1*	64.0*	54.0†	43.0†	56.0†	69.0†
Male (%)	90.9	69.2	84.2	62.5	52.7	66.7	78.9	67.9	75.0	83.9
Risk factors	Smoking 90% Alcohol use 90% COPD 45% DM 36%	Alcohol use 30.8% Malignancy 38.5%; Cerebrovascular disease 30.8% DM 30.8%	Smoking 84.2% COPD 63.2%	DM 37.5% Asthma 37.5% HTN 25%	Smoking 86% Hazardous alcohol use 82% Chronic lung disease 39% DM 27%	Smoking 50% Alcohol use 39% COPD 22% DM 22% Leukopenia 44%	Alcohol use 52.6% Smoking 26.3% Chronic lung disease 21.1%	Smoking 82.1% Hazardous alcohol 71.4% Chronic lung disease 25.0%	Smoking 87.5% Chronic alcohol 75.0%	Smoking 83.9% Chronic lung disease 58.1% DM 38.7% HTN 83.9%
Septic shock (%)	36.4	84.6	57.9	75.0	63.4	55.6	NR	35.7	100.0	38.7
Empiric therapy active (%)	NR	NR	31.6	0.0	100.0	61.1	NR	60.7	0.0	16.0
Mortality (%)‡	64.0	62.0	57.8§	62.5	11‖	61.1	42.1	28.6	62.5	41.9
Seasonality	Wet-season predominant (82% Nov-Mar)	Warm/humid months predominance (85% Apr-Oct)	NR	Warmer months Jun-Sep (≈71%); none Oct-Feb	Wet-season predominant (≈83-88%)	Rainy-season predominance (not statistically significant)	Summer/warm-months predominance; winter rare	Wet season 64.3% (dry 35.7%); no severity/mortality difference by season	Rainy-season predominant (≈75%; rainy Nov-Apr; *P* = .06)	No seasonal predominance (wet vs dry months)

Ab = *Acinetobacter baumannii*, CA-AB = community-acquired *A. baumannii*, CAP = community-acquired pneumonia, COPD = chronic obstructive pulmonary disease, DM = diabetes mellitus, HA-AB = hospital-acquired *A. baumannii*, HTN = hypertension, NR = not reported.

* Reported as mean.

† reported as median.

‡ in-hospital mortality except where footnoted.

§ 30-day mortality.

‖ both 28- and 90-day mortality.

Across the 9 comparative cohorts identified in our review (Table 3), male predominance was consistent, ranging from 52.7% in Australia to 90.9% in earlier Australian series. A recurring finding across cohorts is that CA-AB pneumonia disproportionately affects patients with host or exposure risk factors, particularly smoking, chronic lung disease, diabetes, and hazardous alcohol use, and often presents as rapidly progressive severe CAP. Our cohort aligns closely with this pattern (smoking 83.9%, chronic lung disease 58.1%, diabetes 38.7%). However, the Vietnam cohort appears older than many previously reported CA-AB cohorts, suggesting that in an urban Vietnamese referral population, cumulative exposures and multimorbidity in later life may play a larger role.

Classic descriptions from tropical and island settings suggest wet-season predominance, but more recent evidence shows marked heterogeneity by geography and local ecology. In Ho Chi Minh City, Vietnam, we observed no significant seasonal pattern: cases were nearly evenly split between dry and rainy seasons, and the month-adjusted case rate was similar (1.07 vs 0.71 cases/month; *P* = .261). This differs from wet-season peaks reported in tropical northern Australia,^[7]^ and Singapore,^[2]^ but is consistent with cohorts from North Queensland,^[18]^ Thailand^[3]^ and Réunion Island,^[8]^ where cases occurred across both seasons without worse outcomes in the dry season. Together, these findings suggest that seasonality is context-specific rather than uniformly driven by rainfall or humidity. Possible explanations include urban microclimates, exposure patterns that are not tightly coupled to rainfall, and a colonization-to-infection lag. This is supported by reports of wet-season pharyngeal carriage among at-risk drinkers and by year-round case occurrence without clear dry-season severity penalties in North Queensland.^[18]^ Clinicians should therefore maintain a high index of suspicion year-round and base early empiric coverage on risk factors and severity rather than season alone.

Although CA-AB pneumonia is uncommon, it is frequently fulminant. In our cohort, non-survivors more often required direct RICU admission and had a stronger systemic inflammatory response, particularly higher CRP levels. These findings align with the fulminant presentation described by Leung et al^[6]^ and are consistent with prior reports linking septic shock,^[1,2,18]^ leukocyte abnormalities,^[1]^ and a greater systemic inflammatory response^[18]^ with increased mortality. Across published cohorts, presentation severity is consistently high and outcomes are poor (Table 3); septic shock was common (around 35%–100% where reported). In our cohort, nearly half met the ATS/IDSA severe-CAP criteria, and in-hospital mortality was 41.9%. This mortality was similar to that reported in North America (42.1%),^[22]^ but lower than rates reported in Thailand (61.1%),^[3]^ Singapore (62.5%),^[2]^ and the Réunion cohort (62.5%).^[8]^

Timely, active empiric therapy is therefore crucial, yet empiric selection is complicated by *A. baumannii’s* propensity for multidrug resistance, even in community-onset disease.^[18]^ The species intrinsically carries chromosomal AmpC (ADC) and an OXA-51-like β-lactamase.^[23,24]^ When AmpC is upregulated, third-generation cephalosporins are often ineffective.^[25]^ ESBLs such as PER or VEB can occur, but they are acquired, not intrinsic to the species.^[26]^ In our cohort, resistance to carbapenems and third-generation cephalosporins was approximately 70% or higher, while aminoglycosides demonstrated the greatest in vitro activity among the agents tested (amikacin 50%, tobramycin 46%, gentamicin 36%). This resistance profile appears more extreme than earlier CA-AB series from other countries and may reflect the broader escalation of multidrug-resistant Gram-negative infections in Southeast Asia.^[27]^ Previous genomic studies from some endemic settings have described CA-AB lineages as distinct from healthcare-associated clones and generally less extensively resistant.^[18,21]^ In contrast, our findings suggest that highly resistant *A. baumannii* can also present as community-onset pneumonia in our setting. Several context-specific factors may contribute to this pattern. First, Vietnam has high community antibiotic consumption with widespread non-prescription use (90%–97% in surveys^[28,29]^), and substantial overall antibiotic use,^[30,31]^ which may promote selection and carriage of resistant Gram-negative organisms in the community. Second, national surveillance shows a high burden of carbapenem-resistant *A. baumannii*, with detection of identical or closely related strains in both hospital and rural settings, raising concern for transmission between these environments.^[32]^ Because genomic typing was not performed in our study, we cannot determine whether the resistant phenotype observed reflects community selection, transmission between healthcare and community settings, unrecognized healthcare exposure, or a combination of these mechanisms.

Across cohorts in Table 3, higher rates of early active therapy generally coincided with lower mortality, whereas inactive initial regimens coincided with higher mortality: Davis et al reported 100% active empiric therapy with 11% mortality.^[7]^ Similarly, the Riddles et al cohort, where carbapenem or gentamicin was administered within 6 hours of admission, achieved 60.7% active empiric coverage, with a mortality rate of 28.6%.^[18]^ In contrast, the Singaporean cohort (Ong et al), where 0% of empiric regimens were active, mortality reached 62.5%.^[2]^ In our study, only 16.0% of initial regimens were active and mortality was 41.9%. While these between-study comparisons are observational and may be confounded by differences in host factors, inclusion criteria, and available agents, together they support the biologically plausible concern that delayed active therapy contributes to poor outcomes in this syndrome.

Current CAP guidelines from the ATS/IDSA and the European Respiratory Society,^[9,33]^ prioritize a *β*-lactam plus macrolide or a respiratory fluoroquinolone and do not recommend aminoglycoside monotherapy, given limited pulmonary penetration as monotherapy.^[34]^ In our cohort, standard empiric regimens were frequently inactive against CA-AB pneumonia, while aminoglycosides demonstrated comparatively greater in vitro activity than the other antimicrobial classes evaluated, although absolute susceptibility remained suboptimal (gentamicin 36%, tobramycin 46%, amikacin 50%). Together with their limited pulmonary penetration and risks of nephrotoxicity and ototoxicity, these findings do not support aminoglycoside monotherapy or routine empiric use. Nevertheless, in resource-constrained settings where preferred contemporary anti-*A. baumannii* agents are unavailable or inaccessible, the potential role of susceptibility-confirmed aminoglycosides as components of combination or salvage therapy warrants further evaluation.

Given that carbapenem-resistant *A. baumannii* is designated as a World Health Organization (WHO) critical priority pathogen,^[35]^ local resistance patterns and antimicrobial availability are particularly important when translating international guidance into clinical practice. Our findings emphasize the need for regular local antimicrobial resistance surveillance to inform empiric protocols, support appropriate antimicrobial use, and improve management of this high-risk population. These findings also support periodic review of institutional antimicrobial susceptibility-testing panels, particularly for suspected multidrug-resistant or carbapenem-resistant *A. baumannii*. Future prospective studies should incorporate more comprehensive susceptibility testing whenever feasible.

At our hospital, this therapeutic gap is particularly relevant because the standard CAP policy prioritizes *β*-lactam-based therapy combined with macrolides or respiratory fluoroquinolones, while routine initial anti-A. baumannii coverage is not recommended for most CAP cases. Our findings suggest that this approach is appropriate from an antimicrobial-stewardship perspective for general CAP but may be insufficient for a small subgroup with fulminant severe CAP and risk factors for CA-AB pneumonia. A locally defined escalation pathway, guided by early microbiological sampling and hospital antibiogram data, may help balance timely active therapy against unnecessary broad-spectrum antibiotic use.

In southern Vietnam, regional CAP pathways could in principle incorporate a locally defined “possible CA-AB” branch for patients presenting with fulminant severe CAP or septic shock, informed by local surveillance and facility antibiograms. Such an approach should be considered hypothesis-generating and formally evaluated prospectively before wider implementation. Key elements would include early microbiological sampling in severe CAP and incorporation of local antibiogram data and validated risk factors into empiric decision-making, consideration of additional coverage based on local resistance patterns in carefully selected severe cases, and mandatory reassessment with prompt de-escalation once microbiological results are available. In settings where preferred contemporary agents are unavailable, prospective studies should specifically evaluate treatment strategies applicable to resource-constrained environments, including the role of susceptibility-guided older agents such as aminoglycosides.

Polymyxins are another potential option for suspected or confirmed carbapenem-resistant *A. baumannii* in resource-limited settings, but their clinical use requires careful consideration: observational data suggest that IV colistin with a loading dose may improve survival and clinical/microbiological response but increases nephrotoxicity risk, warranting close renal monitoring,^[36]^ while adjunctive nebulized colistin has not shown a clear survival benefit over IV therapy alone and should therefore be used cautiously based on local feasibility and practice.^[37]^

This study has several limitations. First, the retrospective, single-center design at a large tertiary referral hospital limits generalizability and likely introduces selection bias favoring severe illness, multimorbidity, and multidrug-resistant organisms, potentially inflating reported ICU utilization and mortality. Furthermore, because some outpatient healthcare exposures, including prior clinic visits, home intravenous therapy, and over-the-counter antimicrobial use, were not systematically captured, unrecognized healthcare exposure and consequent misclassification cannot be excluded. Second, reliance on lower-respiratory cultures (sputum/tracheal aspirate) means that colonization cannot be fully excluded despite our use of sputum quality criteria and compatible clinical–radiographic findings, a known limitation of non-sterile specimens. Third, the small sample size warrants cautious interpretation of our findings. Larger studies are needed to confirm the generalizability of the clinical characteristics, resistance patterns, and outcomes described here. Fourth, the antimicrobial susceptibility panel was incomplete. Susceptibility to polymyxins/colistin, tigecycline, minocycline, cefiderocol, and sulbactam-durlobactam was not routinely tested or recorded during the study period. Given the high level of resistance to the conventional agents tested, the absence of susceptibility data for these additional anti-*A.* baumannii agents substantially limits our ability to characterize the full range of therapeutic options and precludes firm treatment recommendations based solely on this dataset. Moreover, susceptibility testing was not performed uniformly across agents. Finally, the lack of genotypic characterization precluded assessment of strain relatedness and community versus hospital lineages. Future research should prioritize prospective, multicenter surveillance, ideally utilizing case-control designs, to better quantify disease burden, seasonality, and risk factors distinguishing CA-AB pneumonia from other pneumonia etiologies. Where feasible, studies should integrate molecular epidemiology with environmental sampling to identify community reservoirs and better distinguish true community acquisition from healthcare-associated spillovers.

## 5. Conclusion

CA-AB pneumonia in southern Vietnam was characterized by severe disease, high levels of antimicrobial resistance, frequent mismatch of initial empiric therapy, and high in-hospital mortality. Resistance was high among routinely tested antimicrobial agents, whereas aminoglycosides demonstrated comparatively greater but still suboptimal in vitro activity. The combination of extensive antimicrobial resistance and a substantial early therapeutic gap underscores the importance of early microbiological diagnosis, comprehensive susceptibility testing, locally informed empiric strategies, and antimicrobial stewardship. In resource-constrained settings where preferred contemporary anti-*A. baumannii* agents are unavailable, susceptibility-guided use of older agents, including aminoglycosides as components of combination therapy, may warrant further prospective evaluation. Prospective multicenter studies incorporating comprehensive susceptibility testing, rapid diagnostics, and genomic characterization are needed to better define treatment strategies and clarify the epidemiology of CA-AB pneumonia.

## Acknowledgements

We thank the Respiratory Department of Nhan Dan Gia Dinh Hospital for their support.

## Author contributions

**Conceptualization:** Thu-Huong Le-Thi, Quoc-Khanh Tran-Le, Hang Nguyen-Thi-Thuy, Nguyen Tien Huy, Tuong Tran-Thi-Thuy.

**Data curation:** Thu-Huong Le-Thi, Quoc-Khanh Tran-Le, Lam Nguyen-Ho, Bao Tran-Gia, Phuong Huynh-Thi-Thanh, Tuong Tran-Thi-Thuy.

**Formal analysis:** Thu-Huong Le-Thi, Quoc-Khanh Tran-Le, Lam Nguyen-Ho, Bao Tran-Gia, Hang Nguyen-Thi-Thuy, Phuong Huynh-Thi-Thanh, Nguyen Tien Huy, Tuong Tran-Thi-Thuy.

**Investigation:** Thu-Huong Le-Thi, Quoc-Khanh Tran-Le, Hang Nguyen-Thi-Thuy, Nguyen Tien Huy, Tuong Tran-Thi-Thuy.

**Methodology:** Thu-Huong Le-Thi, Quoc-Khanh Tran-Le, Lam Nguyen-Ho, Bao Tran-Gia, Hang Nguyen-Thi-Thuy, Phuong Huynh-Thi-Thanh, Nguyen Tien Huy, Tuong Tran-Thi-Thuy.

**Visualization:** Quoc-Khanh Tran-Le, Tuong Tran-Thi-Thuy.

**Project administration:** Tuong Tran-Thi-Thuy.

**Resources:** Tuong Tran-Thi-Thuy.

**Software:** Tuong Tran-Thi-Thuy.

**Supervision:** Tuong Tran-Thi-Thuy.

**Writing – original draft:** Thu-Huong Le-Thi, Quoc-Khanh Tran-Le, Lam Nguyen-Ho, Bao Tran-Gia, Hang Nguyen-Thi-Thuy, Phuong Huynh-Thi-Thanh, Nguyen Tien Huy, Tuong Tran-Thi-Thuy.

**Writing – review & editing:** Thu-Huong Le-Thi, Quoc-Khanh Tran-Le, Lam Nguyen-Ho, Bao Tran-Gia, Hang Nguyen-Thi-Thuy, Phuong Huynh-Thi-Thanh, Nguyen Tien Huy, Tuong Tran-Thi-Thuy.
